# Tornadic Shear Stress Induces a Transient, Calcineurin-Dependent Hypervirulent Phenotype in Mucorales Molds

**DOI:** 10.1128/mBio.01414-20

**Published:** 2020-06-30

**Authors:** Sebastian Wurster, Alexander M. Tatara, Nathaniel D. Albert, Ashraf S. Ibrahim, Joseph Heitman, Soo Chan Lee, Amol C. Shetty, Carrie McCracken, Karen T. Graf, Antonios G. Mikos, Vincent M. Bruno, Dimitrios P. Kontoyiannis

**Affiliations:** a Department of Infectious Diseases, Infection Control and Employee Health, The University of Texas M. D. Anderson Cancer Center, Houston, Texas, USA; b Department of Bioengineering, Rice University, Houston, Texas, USA; c Los Angeles Biomedical Research Institute at Harbor-UCLA Medical Center, Los Angeles, California, USA; d David Geffen School of Medicine at ULCA, Los Angeles, California, USA; e Departments of Molecular Genetics and Microbiology, Pharmacology and Cancer Biology, and Medicine, Duke University Medical Center, Durham, North Carolina, USA; f South Texas Center of Emerging Infectious Diseases, Department of Biology, University of Texas at San Antonio, San Antonio, Texas, USA; g The Institute for Genome Sciences, University of Maryland, Baltimore, Maryland, USA; Universidade de Sao Paulo

**Keywords:** mucormycosis, virulence, mechanobiology, trauma, stress response

## Abstract

Trauma-related necrotizing myocutaneous mucormycosis (NMM) has a high morbidity and mortality in victims of combat-related injuries, geometeorological disasters, and severe burns. Inspired by the observation that several recent clusters of NMM have been associated with extreme mechanical forces (e.g., during tornados), we studied the impact of mechanical stress on Mucoralean biology and virulence in a Drosophila melanogaster infection model. In contrast to other experimental procedures to exert mechanical stress, tornadic shear challenge (TSC) by magnetic stirring induced a hypervirulent phenotype in several clinically relevant Mucorales species but not in *Aspergillus* or *Fusarium*. Whereas fungal growth rates, morphogenesis, and susceptibility to noxious environments or phagocytes were not altered by TSC, soluble factors released in the supernatant of shear-challenged R. arrhizus spores rendered static spores hypervirulent. Consistent with a rapid decay of TSC-induced hypervirulence, minimal transcriptional changes were revealed by comparative RNA sequencing analysis of static and shear-challenged Rhizopus arrhizus. However, inhibition of the calcineurin/heat shock protein 90 (hsp90) stress response circuitry by cyclosporine and tanespimycin abrogated the increased pathogenicity of R. arrhizus spores following TSC. Similarly, calcineurin loss-of-function mutants of Mucor circinelloides displayed no increased virulence capacity in flies after undergoing TSC. Collectively, these results establish that TSC induces hypervirulence specifically in Mucorales and point out the calcineurin/hsp90 pathway as a key orchestrator of this phenotype. Our findings invite future studies of topical calcineurin inhibitor treatment of wounds as an adjunct mitigation strategy for NMM following high-energy trauma.

## Abstract (precis)

Given the limited efficacy of current medical treatments in trauma-related necrotizing mucormycosis, there is a dire need to better understand the Mucoralean pathophysiology in order to develop novel strategies to counteract fungal tissue invasion following severe trauma. Here, we describe that tornadic shear stress challenge transiently induces a hypervirulent phenotype in various pathogenic Mucorales species but not in other molds known to cause wound infections. Pharmacological and genetic inhibition of calcineurin signaling abrogated hypervirulence in shear stress-challenged Mucorales, encouraging further evaluation of (topical) calcineurin inhibitors to improve therapeutic outcomes of NMM after combat-related blast injuries or violent storms.

## INTRODUCTION

Necrotizing myocutaneous invasive mold infections following severe trauma represent a life-threatening disease with high morbidity and mortality (1–4). A variety of molds have been implicated as causative agents, with fungi belonging to the order Mucorales predominating as the most common and devastating cause (3, 4). These emerging fungal pathogens affect an expanding population of hosts and are characterized by innate resistance to many antifungals, broad geographic and environmental distribution, and high virulence (5–8). Although immunocompromised patients are at highest risk for the development of mucormycosis (5–8), immunocompetent individuals are prone to necrotizing myocutaneous mucormycosis (NMM) when incurring penetrating trauma (2, 9–12), combat-related wounds (13–15), or burn injuries (1).

Interestingly, several clusters of NMM have been observed after trauma events in settings of extreme mechanical forces. Specifically, NMM has emerged in military personnel suffering wound infections after blast injuries from improvised explosive devices in Afghanistan and Iraq, and the recovery of Mucorales from wound cultures was frequently associated with recurrent tissue necrosis (15). Moreover, a cluster of NMM cases due to Apophysomyces trapeziformis, an uncommon agent of mucormycosis, was reported after the 2011 EF-5 Joplin tornado, causing considerable mortality and necessitating aggressive debridement and complex reconstruction among survivors (10). NMM cases were also reported after the 2004 Indian Ocean tsunami (9, 16). Given that NMM is encountered after high-energy events, we hypothesized that mechanical stress challenge may alter the virulence traits and eventually result in increased pathogenicity of Mucorales molds.

Studies in bacteriology suggest that prokaryotic pathogens experience and sense a variety of mechanical events, including shear forces that can modulate motility, surface adhesion, and biofilm formation (17, 18). There is increasing evidence that shear forces also influence eukaryotic cell behavior, proliferation, and signaling (19, 20), and previous reports have highlighted that cascades involved in environmental stress response serve crucial roles in controlling fungal morphogenesis and pathogenic capacity (21, 22). However, the specific link between mechanical stress and fungal virulence is as yet uncharacterized. To address this gap of knowledge in the context of trauma-related NMM, we studied the impact of tornadic shear forces on the morphogenesis, transcriptional signatures, and pathogenicity of Mucorales. Using a Drosophila melanogaster mucormycosis model that has been validated to recapitulate key virulence attributes of human infections (23, 24), we found a transient, hypervirulent phenotype in shear-challenged Mucorales but not in other common opportunistic molds. Our findings further reveal that shear force-induced hypervirulence in Mucorales relies on the calcineurin/heat shock protein 90 (hsp90) pathway, giving rise to new potential avenues of therapeutic interventions in NMM following combat injuries or geometeorological disasters.

## RESULTS

### Tornadic shear challenge induces a unique, transient, hypervirulent phenotype of Mucorales.

At first, we compared the influence of different tornadic shear challenge (TSC) procedures on the *in vivo* pathogenicity of Rhizopus arrhizus, the most common cause of mucormycosis (25). Spore suspensions were either centrifuged, vortexed, or stirred with a magnetic stir rod for 30 min. Wild-type (WT) D. melanogaster flies infected with static R. arrhizus spores showed 7-day survival rates of 37 to 41% (see Fig. S1A to C in the supplemental material), consistent with our previous findings (23, 26). Neither centrifuged (Fig. S1A) nor vortexed (Fig. S1B) spores elicited increased mortality in infected flies compared to static spores (*P* = 0.89 and 0.91, respectively). In contrast, magnetic stirring of R. arrhizus spores led to near universal lethality of flies in 7 days (6% survival versus 41%, *P* < 0.001) and reduced the median survival time of infected flies from 5 to 2 days (Fig. S1C), suggesting enhanced fungal pathogenicity. Therefore, magnetic stirring was used to simulate TSC in all subsequent experiments.

10.1128/mBio.01414-20.1 FIG S1 Comparative evaluation of laboratory procedures for shear stress exposure of Mucorales spores. R. arrhizus Ra-969 spore suspensions (10^7^/ml) were exposed to TSC by centrifugation at 6,000 rpm (A), vortexing (B), or magnetic stirring at ∼1,100 rpm (C) for 30 min. Controls were kept in static culture for the same time. WT D. melanogaster flies were pricked with a needle dipped into the spore solutions. Two independent experiments were performed with a total of 46 to 52 flies per condition. Survival curves were compiled from aggregated results (assessed using a log rank test). Error bars represent inter-replicate SD. Download FIG S1, TIF file, 2.7 MB. Copyright © 2020 Wurster et al. 2020 Wurster et al. This content is distributed under the terms of the Creative Commons Attribution 4.0 International license.

**FIG 1:**
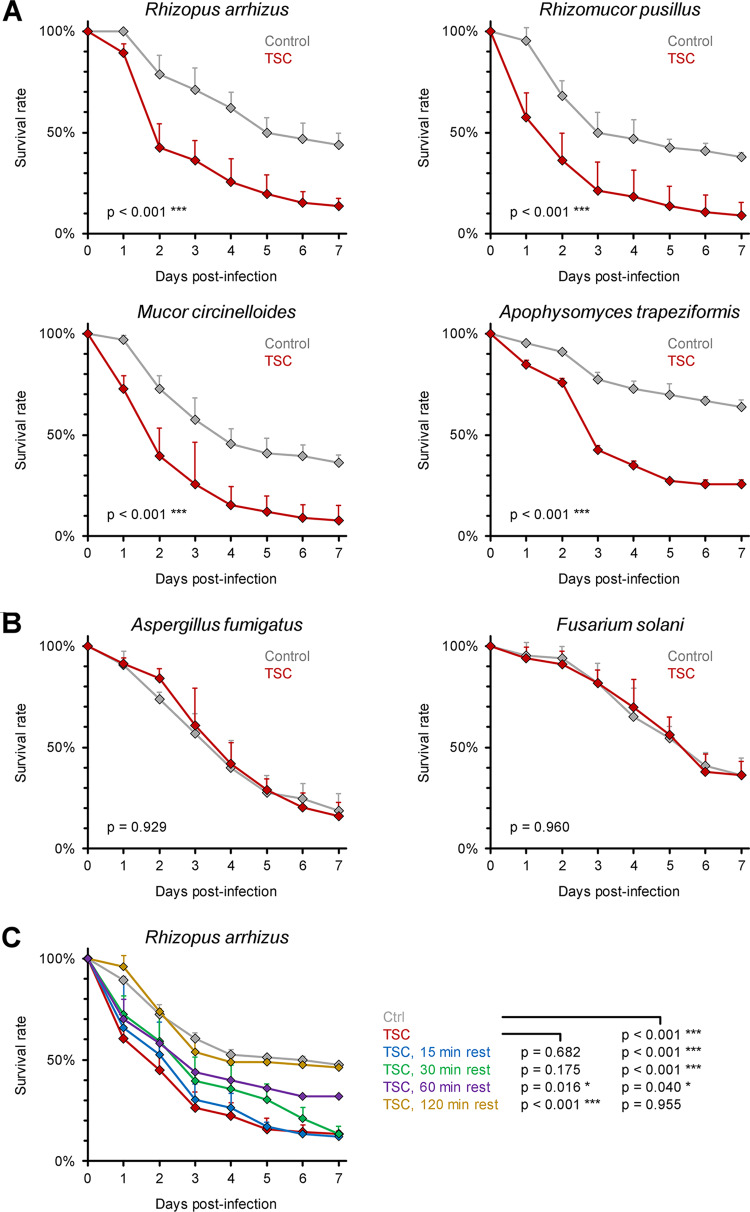
Tornadic shear challenge transiently increases the pathogenicity of Mucorales but not Ascomycetes. (A and B) Spore suspensions (10^7^/ml) of Mucorales isolates R. arrhizus Ra-749, *R. pusillus* Rp-449, *M. circinelloides* Mc-518, and *A. trapeziformis* CBS 125534 (A), as well as Ascomycetes isolates A. fumigatus Af-293 and F. solani Fs-001 (B), were subjected to TSC by magnetic stirring for 30 min or kept in static culture (Control). D. melanogaster flies were infected by pricking with a needle dipped into the spore suspensions. WT flies were used for Mucorales and F. solani, whereas A. fumigatus infections were performed in *Tl^r632^/Tl^I-RXA^* mutant flies since *Aspergillus* infections are nonlethal in WT flies. For each pathogen, three independent experiments were performed, with a total of 65 to 69 flies per condition. (C) WT flies were infected with R. arrhizus Ra-749 (10^7^/ml) either immediately after termination of TSC or after a 15- to 120-min resting period. The nonchallenged control was inoculated within 5 min of the nonrested TSC cohort. Three independent experiments were performed with a total of 76 flies per condition. For all panels, survival curves were compiled from aggregated results (assessed using a log rank test). Error bars represent inter-replicate standard deviations (SD).

In order to evaluate the generalizability of TSC-induced hypervirulence in Mucorales, we tested additional clinical isolates of R. arrhizus, Rhizomucor pusillus, and Mucor circinelloides. For all three strains, TSC led to a precipitous decline in 7-day survival rates of infected WT flies from 36 to 44% to 8 to 14% (*P* < 0.001, Fig. 1A). Interestingly, the increase in virulence after TSC was particularly pronounced in *A. trapeziformis*, with a 26% 7-day survival rate of infected flies compared to 64% for flies infected with static spores (*P* < 0.001, Fig. 1A), concordant with the dominance of *A. trapeziformis* after high-energy trauma (1, 10, 27). However, as it is challenging to obtain large quantities of *Apophysomyces* spores, we used R. arrhizus as a model organism for further investigations of TSC-induced pathogenicity. In contrast to Mucorales, the pathogenicity of opportunistic Ascomycetes molds Aspergillus fumigatus and Fusarium solani was not altered by magnetic stirring (Fig. 1B), indicating that TSC induces increased virulence specifically in Mucorales.

To evaluate whether increased virulence following TSC was dependent on the infecting spore concentration, we pricked flies with a needle immersed in a range of different R. arrhizus spore inocula (10^4^, 10^6^, or 10^8^/ml) that underwent TSC by magnetic stirring or remained under static conditions. Expectedly, baseline mortality without TSC was inoculum-dependent, with 7-day-survival rates of 49% (10^4^ spores/ml), 39% (10^6^/ml), and 36% (10^8^/ml), respectively (Fig. S2). For all inocula tested, *Rhizopus* infection following TSC led to significantly elevated fly mortality (*P* < 0.001), with essentially identical deltas in 7-day mortality rates (28 to 31%). Collectively, these data suggest that enhanced pathogenicity of Mucorales after TSC is a strain-, species-, and inoculum-independent phenomenon.

10.1128/mBio.01414-20.2 FIG S2 TSC-induced hypervirulence is encountered across a wide range of spore concentrations. R. arrhizus Ra-969 spore suspensions were prepared at concentrations of 10^4^ (A), 10^6^ (B), and 10^8^ (C) spores per ml. Spores were subjected to TSC by magnetic stirring or kept in static culture for the same time (control). WT D. melanogaster flies were pricked with a needle dipped into the spore solutions. Three independent experiments were performed with a total of 69 to 78 flies per condition. Aggregated results were used for survival curves (assessed using a log rank test). Error bars represent inter-replicate SD. Download FIG S2, TIF file, 1.4 MB. Copyright © 2020 Wurster et al. 2020 Wurster et al. This content is distributed under the terms of the Creative Commons Attribution 4.0 International license.

We then assessed the durability of the hypervirulent phenotype after TSC exposure of R. arrhizus. In line with our earlier results (Fig. 1A), flies pricked immediately after magnetic stirring of the fungal inoculum suspension exhibited significantly increased 7-day mortality compared to the static control (87% versus 53%, *P* < 0.001, Fig. 1C). However, enhanced pathogenicity in flies decayed rapidly with increasing resting periods after TSC. A 60-min resting step following TSC exposure of R. arrhizus spores significantly reduced fly mortality and the survival curves reverted back to the static control after 120 min post-TSC resting (Fig. 1C), indicating that the increased pathogenicity of Mucorales after exposure to TSC is transient.

### Soluble factors contribute to Mucoralean hypervirulence following TSC.

**FIG 2:**
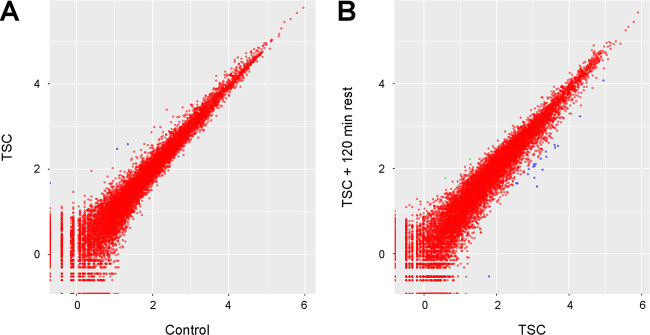
Tornadic shear challenge does not significantly alter the transcriptome of R. arrhizus. (A and B) Scatterplots comparing the expression of each gene in R. arrhizus Ra-749 spores exposed to TSC or not (Control) (A), as well as spores exposed to TSC and then allowed to rest for 120 min or not (B). Values represent the log-transformed mean-normalized read count for each gene. Red dots indicate an FDR >0.05 (deemed not differentially expressed). Blue or green dots indicate differentially expressed genes (FDR <0.05).

To understand the molecular mechanisms underlying the increased virulence following TSC, we performed RNA sequencing (RNA-seq) on R. arrhizus spores that were either kept in static culture, exposed to TSC, or exposed to TSC and then allowed to rest for 120 min. We defined differentially expressed genes as those with a false discovery rate (FDR) of <0.05 between experimental groups. Despite the significant sequencing depth coverage that we obtained (38.1 ± 3.62 million reads per sample), we observed that R. arrhizus mounted a minimal transcriptional response to TSC (Fig. 2A) and also to the rest following TSC (Fig. 2B). In fact, only three genes were differentially expressed between shear-challenged and static spores. Furthermore, only 22 genes were differentially expressed between the spores that were exposed to TSC and those that were allowed to rest for 120 min following shear challenge. All of the differentially expressed genes are uncharacterized and annotated as hypothetical proteins. Taken together, these results suggest that R. arrhizus does not mount a robust transcriptional response to TSC and that the increased virulence is likely not the result of transcriptional upregulation of virulence genes.

**FIG 3:**
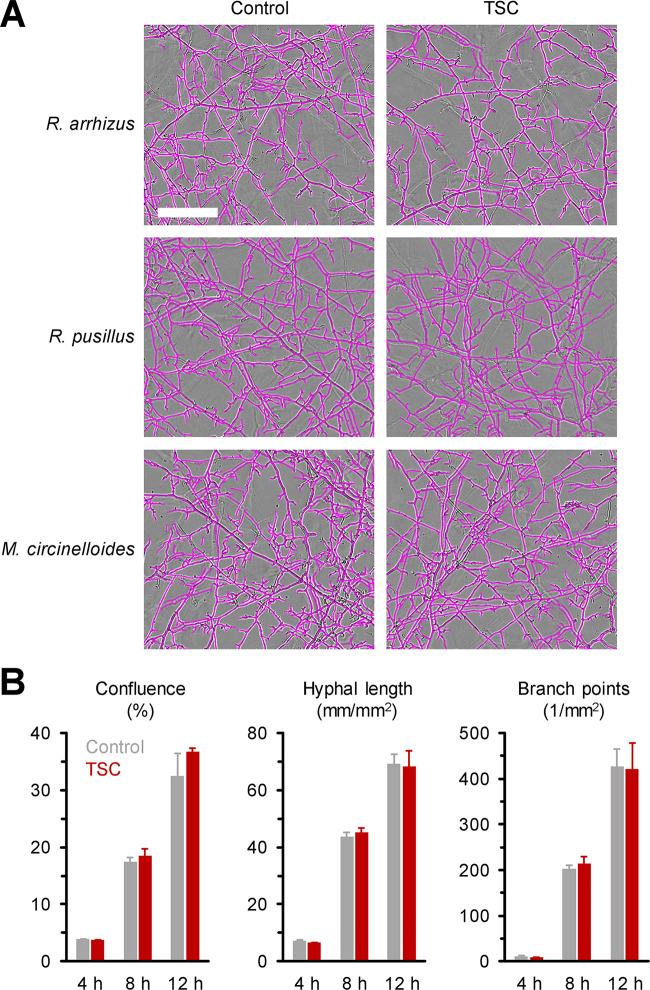
TSC does not alter the morphology and mycelial expansion kinetics of Mucorales *in vitro*. Rhizopus arrhizus Ra-749, Rhizomucor pusillus Rp-449, and Mucor circinelloides Mc-518 spore suspensions (10^7^/ml) were exposed to TSC by magnetic stirring for 30 min or kept in static culture (Control). Spore suspensions were subsequently diluted to 1 × 10^3^/ml in RPMI plus 2% glucose, and 200-μl aliquots were added to 96-well flat-bottom plates (200 spores per well). Plates were imaged hourly in the IncuCyte ZOOM time lapse microscopy system (37°C). (A) Representative images after 12 h of culture are shown. Scale bar, 200 μm. (B) NeuroTrack (NT) and basic analyzer (BA) processing definitions were used to determine mycelial confluence (BA), total hyphal length (NT), and branch point numbers (NT) of Ra-749 after 4, 8, and 12 h of culture. Means plus the SD (*n* = 3) are shown.

Therefore, we conducted an array of phenotypic assays to further understand the features of TSC-induced hypervirulence. Employing the recently adapted IncuCyte NeuroTrack time-lapse imaging approach (28), we tested whether TSC results in accelerated growth or hyphal filamentation of Mucorales. However, mycelial morphology (Fig. 3A), confluence, hyphal length, and branch point numbers (Fig. 3B and data not shown) remained unaffected by TSC in three different Mucorales isolates, indicating that hypervirulence is not a result of altered mycelial expansion and/or morphogenesis.

Since previous studies suggested that fungal stress adaptation can induce resistance to subsequent stress events (21, 22), we further used the NeuroTrack assay to determine the impact of TSC on the Mucoralean tolerance of oxidative stress (peroxide and H_2_O_2_). In all three species tested, shear-challenged spores did not have increased resistance to subsequent peroxide exposure compared to static controls (Fig. S3A). Similarly, no significantly different hyphal length endpoints were found between shear-challenged and static Mucorales spores subsequently exposed to subinhibitory peroxide concentrations (Fig. S3A). In addition, simultaneous exposure to TSC and the highest subinhibitory peroxide concentration (1 mM) had no change on mycelial expansion compared to Mucorales spores exposed to 1 mM H_2_O_2_ in static culture (data not shown). To corroborate these data with a different stressor, we exposed shear-challenged and control spores to serial dilutions of amphotericin B and posaconazole, resulting in nondifferential MICs (Fig. S3B). Collectively, these data suggest that TSC does not affect the ability of Mucorales to survive and form expansive mycelium in noxious *in vitro* environments.

10.1128/mBio.01414-20.3 FIG S3 The susceptibility of Mucorales to subsequent oxidative and antifungal stress remains unchanged after TSC. Rhizopus arrhizus Ra-749, Rhizomucor pusillus Rp-449, and Mucor circinelloides Mc-518 spore suspensions (10^7^/ml) were exposed to TSC by magnetic stirring for 30 min or kept in static culture (Control). Spore suspensions were subsequently diluted to 2 × 10^3^/ml in RPMI plus 2% glucose. (A) Portions (100 μl) of the suspensions were added to 96-well flat-bottom plates (200 spores per well) containing 100 μl of RPMI with serial dilutions of H_2_O_2_ (final concentration, 0 to 64 mM). Phase images were obtained hourly for 24 h in the IncuCyte ZOOM time lapse microscopy system. NeuroTrack processing definitions were used to determine hyphal length and branch point numbers depending on the H_2_O_2_ concentration and prior TSC exposure. Mean maximum hyphal length and branch point numbers (*n* = 4) observed during the 24-h observation period and SD are shown. (B) Portions (100 μl) of the spore suspensions were mixed with serial dilutions of posaconazole (0.03 to 16 μg/ml) and amphotericin B (0.03 to 16 μg/ml) in 96-well round-bottom plates, and the MICs were determined after 48 h according to CLSI reference methods M38. Two independent replicates were performed. Download FIG S3, TIF file, 1.4 MB. Copyright © 2020 Wurster et al. 2020 Wurster et al. This content is distributed under the terms of the Creative Commons Attribution 4.0 International license.

**FIG 4:**
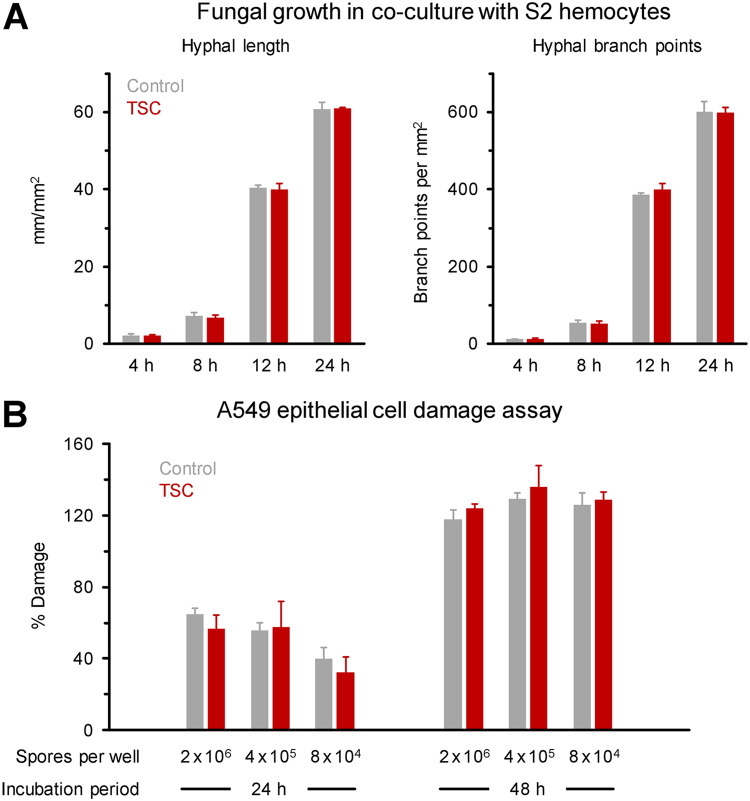
Common virulence traits of Mucorales are unchanged after TSC. (A) Spore suspensions of GFP-expressing R. arrhizus (FTR1-GFP-R. arrhizus, 10^7^/ml) were either exposed to TSC for 30 min or kept under static conditions (Control) and then diluted in complete Schneider’s medium at a concentration of 10^4^/ml. Then, 100-μl aliquots of the suspensions (10^3^ spores) were combined with 10^4^ S2 hemocytes (effector/target ratio of 10:1) diluted in 100 μl of complete Schneider’s medium in a 96-well flat-bottom plate. The plate was incubated in the IncuCyte ZOOM time-lapse microscopy system for 24 h at 28°C. Phase and green fluorescence (400-ms acquisition time) images were obtained hourly. The mycelial length and branch points were quantified by NeuroTrack analysis. Means plus the SD are shown (*n* = 3). (B) A549 alveolar epithelial cells were infected with *R. delemar* 99-880 spores that were subjected to TSC or kept under static conditions. A549 cell damage was quantified by LDH measurement, as described below. The data are expressed as means + the SD based on three technical replicates.

Since increased resistance to or interference with the host’s phagocytic capacity could present another virulence feature contributing to TSC-induced pathogenicity, we next compared the phagocytic activity of D. melanogaster S2 hemocytes against shear-challenged and static R. arrhizus. S2 cells have considerable similarities with human phagocytic cells and have been previously validated as an *in vitro* system to study cellular immune responses against Mucorales (23). However, cocultures analyzed by IncuCyte NeuroTrack imaging revealed no differential susceptibility of static and shear-challenged R. arrhizus spores to S2 phagocytes, as indicated by comparable mycelial expansion and branching kinetics in the presence of phagocytes, regardless of prior TSC (Fig. 4A). In addition, invasion of host epithelia is regarded as an essential virulence trait in the establishment and progression of mucormycosis (29, 30). Upon coculturing *Rhizopus* spores with A549 epithelial cells for 24 and 48 h, static and TSC-exposed spores elicited comparable release of lactate dehydrogenase (LDH), a surrogate marker for epithelial cell damage (Fig. 4B).

**FIG 5:**
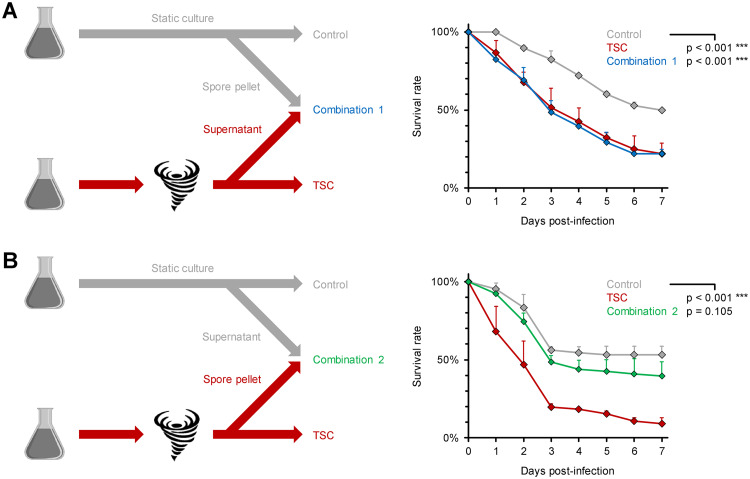
The hypervirulent phenotype of shear-challenged Mucorales is driven by a soluble factor. R. arrhizus Ra-749 spore suspensions (10^7^/ml) were exposed to TSC by magnetic stirring or kept in static culture (control) for 30 min. Additional suspensions (combinations 1 and 2) were prepared by replacing the supernatant from control spores with an equivalent volume of supernatant from TSC-exposed samples (A) or *vice versa* (B). Survival rates of infected flies were monitored for 7 days. A total of 66 to 68 flies per condition were tested in three independent replicates (assessed using a log rank test). Error bars represent inter-replicate SD.

Next, we focused our attention on the possibility that secreted metabolites could contribute to increased pathogenicity in the TSC setting. Interestingly, static R. arrhizus spores resuspended in supernatants from TSC-exposed spores became equally hypervirulent in the fly model as the TSC inoculum, with 7-day survival rates of 22% versus 50% with nonchallenged control spores (*P* < 0.001, Fig. 5A). Inversely, the mortality of flies infected with a mixture of supernatants from static R. arrhizus spores and the TSC-exposed spore pellet was comparable to the unchallenged control and significantly lower than in flies infected with stirred spores (*P* < 0.001, Fig. 5B). Together, these data suggest that soluble factors contribute to increased virulence of TSC-exposed R. arrhizus spores.

### TSC-induced hypervirulence depends on the Mucoralean calcineurin/hsp90 pathway.

**FIG 6:**
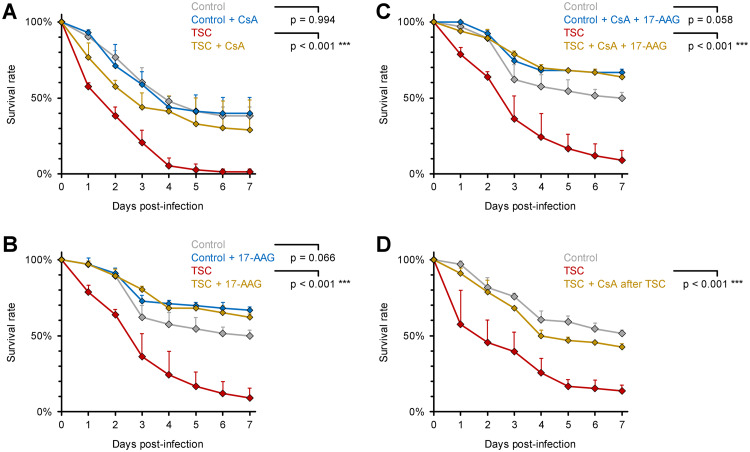
Mucoralean hypervirulence after tornadic shear challenge depends on calcineurin and hsp90. (A to C) R. arrhizus Ra-749 spore suspensions (10^7^/ml) were either prepared in PBS or in PBS supplemented with inhibitors of the calcineurin/hsp90 pathway (100 μg/ml CsA [A], 50 μg/ml 17-AAG [B], or 100 μg/ml CsA + 50 μg/ml 17-AAG [C]). Spores were exposed to TSC by magnetic stirring for 30 min or kept in static culture for the same period (Control). WT D. melanogaster flies were infected by pricking with a needle dipped into the spore suspensions. (D) Flies were infected with static or shear-challenged R. arrhizus Ra-749 spore suspensions (10^7^/ml) supplemented with 100 μg/ml CsA after the TSC procedure or not. For each panel, three independent experiments were performed with a total of 66 to 73 flies per condition. Survival curves were compiled from aggregated results (assessed using a log rank test). Error bars represent inter-replicate SD.

Lastly, we sought to identify cascades governing the hypervirulent phenotype of Mucorales after TSC. As the calcineurin/hsp90 axis has been described as a pivotal driver of fungal adaptation to environmental stress (20, 22, 31, 32), we hypothesized that inhibitors of this pathway may attenuate the impact of TSC on Mucoralean hypervirulence. Indeed, addition of subinhibitory concentrations (100 μg/ml) of the calcineurin inhibitor cyclosporine (CsA) during TSC exposure of R. arrhizus reduced the 7-day mortality of infected flies from 99 to 71% (Fig. 6A and *P* < 0.001), whereas the pathogenicity of static spores was not influenced by CsA. Furthermore, the hsp90 inhibitor tanespimycin (50 μg/ml 17-AAG, Fig. 6B) and its combination with CsA (Fig. 6C) fully reverted the pathogenicity of TSC-exposed spores to the level of the unchallenged control, further underscoring a role of the calcineurin/hsp90 pathway in TSC-induced *in vivo* pathogenicity. Importantly, CsA continued to mitigate TSC-induced hypervirulence of R. arrhizus even when added after the stirring procedure (Fig. 6D and *P* < 0.001), an important finding that indicates potential feasibility of pharmacological interventions to counteract fungal virulence in NMM after high-energy events.

**FIG 7:**
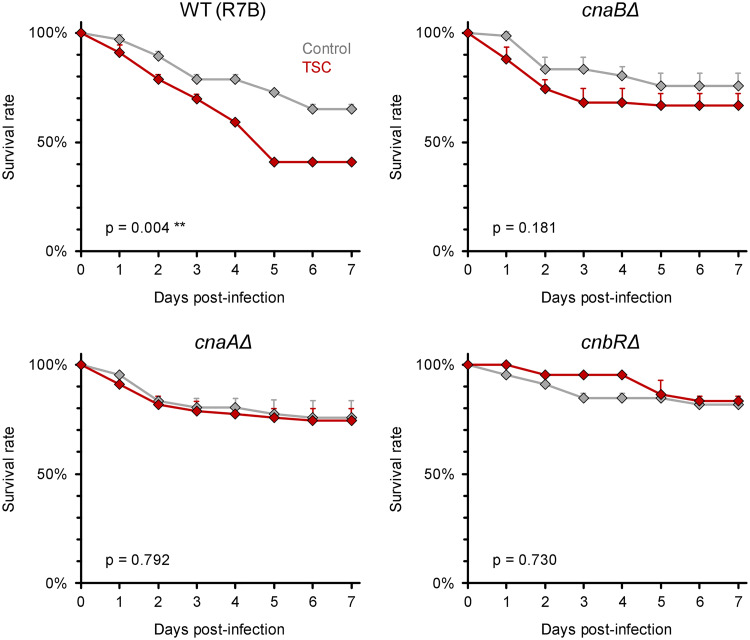
Loss-of-function of calcineurin catalytic or regulatory subunits abrogates TSC-induced hypervirulence in M. circinelloides. Spore suspensions (10^7^/ml) of *M. circinelloides* calcineurin subunit loss-of-function mutants MSL9 (*cnaAΔ*), MSL22 (*cnaBΔ*), MSL8 (*cnbRΔ*), and the isogenic R7B control strain were exposed to TSC by magnetic stirring for 30 min or kept in static culture (control). WT D. melanogaster flies were infected by pricking with a needle dipped into the spore suspensions. For each panel, three independent experiments were performed with a total of 66 flies per strain and condition. Survival curves were compiled from aggregated results (assessed using a log rank test). Error bars represent inter-replicate SD.

To corroborate the critical relevance of the calcineurin/hsp90 axis for TSC-induced hyper-virulence, we employed previously described *M. circinelloides* mutants harboring a loss-of-function of the two calcineurin catalytic A subunits (*cnaAΔ* and *cnaBΔ*) and regulatory B subunit (*cnbRΔ*) (33, 34). Expectedly, an isogenic wild-type control (R7B) strain of *M. circinelloides* displayed significantly enhanced virulence after undergoing TSC, with 7-day survival rates of infected flies dropping from 65% (static control) to 41% (*P* = 0.004, Fig. 7). In contrast, no significant difference in pathogenic capacity was seen between static and shear-challenged spores for all three *M. circinelloides* calcineurin loss-of-function mutants tested (Fig. 7), thus providing further support for a model whereby TSC elicits transient hypervirulence of Mucorales in a calcineurin-dependent way.

## DISCUSSION

Clusters of NMM cases in patients suffering trauma in settings of extreme shear forces such as blast injuries or tornados gave rise to the hypothesis that extreme mechanical forces may impact the virulence of Mucorales. Mimicking TSC *in vitro* by high-speed cyclonical rotation on a magnetic stirrer, we found increased pathogenicity of Mucorales in our fruit fly infection model as documented by excess mortality of infected flies. Interestingly, other types of mechanical forces, such as centrifugation or vortexing, did not result in increased virulence, suggesting that the nature and intensity of physical forces determine differential effects on fungal biology. While different tube/flask sizes used for the three techniques to exert shear stress challenge have likely contributed to the disparate efficacy of these procedures to induce Mucoralean hypervirulence, previous work has established that the tangential flow and velocity fields in a magnetic stirrer are dynamically similar to that of big atmospheric vortices such as tornadoes (35). As the rotational speed and viscous effects in the rotating flow of a common laboratory stirrer are several magnitudes smaller than in a tornado (35), prolonged exposure time (≥30 min) was needed to reliably induce Mucoralean hypervirulence. Although most tornados last for only a few minutes, violent tornados such as the 2011 Joplin tornado can reach path lengths over 100 miles and travel for more than 30 min (36, 37), underlining the physiological relevance of our experimental setting. Nonetheless, there is a possibility that other physical effects of the stirring system that are not encountered in a real-life tornado (e.g., magnetic fields) could have contributed to enhanced pathogenicity. To corroborate broader applicability of our findings and to dissect the impact of different force kinetics on Mucoralean mechanobiology, experimental systems recapitulating combat-related blast injury by air pressure-induced shock waves (38) or miniature explosives (39) could present an exciting direction for future studies.

Unexpectedly, we found that Mucoralean hypervirulence after TSC was not related to changes in fungal proliferation or capacity to cope with adverse conditions such as oxidative stress challenge, antifungal agents, or exposure to phagocytes. In line with these observations, RNA sequencing analysis of R. arrhizus revealed a very low number of significantly differentially expressed transcripts upon TSC. Specifically, no genes that are known or suspected to be immediately linked with Mucoralean virulence such as epithelial invasins, toxins, or proteins related to iron metabolism (40) were differentially expressed after TSC. While the identification of novel virulence factors and interpretation of the few differentially regulated transcripts is complicated by the sparse annotation of the *Rhizopus* genome and very limited experimental characterization of Mucoralean gene functions, the overall minimal transcriptional changes and rapid decay of hypervirulence suggest a role of posttranslational events that would have not been captured by our sequencing approach.

Our findings indicate that Mucoralean hypervirulence after TSC is partly driven by soluble metabolites that are released by shear-challenged spores and can subsequently increase the pathogenicity of static control spores. While struggling to solve the enigma of identifying the causative soluble factors, characterization of the overarching regulatory cascades driving shear stress-induced hypervirulence could provide cues for potential therapeutic targets to improve the detrimental outcomes of NMM after high-energy trauma. Earlier studies of fungal homeostatic stress response following physical, oxidative, and antifungal challenge have revealed the evolutionary conserved calcineurin/hsp90 pathway as a fungal Achilles’ heel due to its role as a multifunctional regulator of cell wall integrity, adaption to adverse environments, and virulence (31–34, 41, 42). Therefore, we hypothesized that this pathway may also be a gatekeeper of the Mucoralean response to TSC. Indeed, single and dual pharmacological inhibition of the calcineurin/hsp90 axis fully reverted TSC-induced hypervirulence of R. arrhizus in fruit flies, and the use of well-defined *M. circinelloides* calcineurin mutants (33, 34) corroborated the pharmacological phenocopy.

While calcineurin homologues are widely encountered throughout eukaryotic kingdoms, including fungi (43), unusually high numbers of calcineurin pathway components were identified in Mucorales and have been implicated in Mucoralean virulence by governing dimorphic transitions and cell wall integrity (33, 34, 39). Inhibition of the calcineurin pathway sensitized Mucorales to azole-induced apoptotic death and led to improved *in vitro* and *in vivo* efficacy of azole antifungals (44–46), highlighting its relevance to Mucoralean stress tolerance. Furthermore, recent reports have revealed noncanonical RNA interference pathways of *M. circinelloides* as posttranscriptional regulators of pathogenesis-related molecular processes including calcineurin pathway components in response to stressful stimuli (34, 47). This mechanism would deserve further characterization in the context of TSC-induced hypervirulence. Importantly, the cited reports primarily described a role of the calcineurin/hsp90 pathway in the maintenance of cellular functionality and pathogenicity following exogenous stress events. In contrast, our observations in the TSC setting suggest that specific environmental stress stimuli can enhance Mucoralean *in vivo* virulence beyond the baseline level encountered under homeostatic conditions in a calcineurin/hsp90-dependent manner.

Calcineurin A has been shown to stress dependently associate with endoplasmic reticulum membranes in Cryptococcus neoformans and has been hypothesized to influence fungal membrane trafficking and protein folding (48). These observations constitute a potential role of calcineurin in the posttranslational stage of protein biosynthesis that could possibly contribute to stress-induced alterations of the Mucoralean secretome. However, comparable attenuation of shear stress-induced hypervirulence by CsA treatment of *Rhizopus* spores before and after TSC suggests that the release of soluble factors during TSC may be not be actively governed by the calcineurin/hsp90 pathway. Consistent with this observation, supernatants from spores that were exposed to TSC in the presence of CsA maintained their capacity to render static spores hypervirulent (data not shown). We therefore favor the hypothesis that the calcineurin/hsp90 axis primarily modulates features of post-TSC pathogenicity in the infected host.

Traditional antifungals perform poorly in NMM, as effective concentrations are difficult to achieve in inflamed or necrotic tissue (49) and the causative fungal agents are frequently multidrug resistant (50). Our observation that TSC-induced hypervirulence can be effectively mitigated by post-TSC CsA treatment would encourage future studies of calcineurin inhibitor therapy as an adjunct strategy in trauma-related NMM after high-energy events. Whereas systemically applied calcineurin inhibitors can potentiate immune paralysis, cause drug-drug interactions with many commonly applied antifungals (51), and exert adverse effects on wound healing (52), topical administration of calcineurin inhibitors to the wound environment could present an appealing alternative. While release kinetics in infected tissue remain to be investigated, modern formulations for topical CsA therapy can achieve tissue concentrations above 100 μg/ml within few hours of dermal application to lesioned skin (53), underscoring the translational relevance of the CsA concentration selected for our experiments.

This study has some limitations. D. melanogaster has been well validated as a rapid, genetically amenable tool to study the virulence and immunopathogenesis of filamentous fungi (23, 24), and there is robust evidence that Mucorales employ common virulence strategies to invade evolutionarily disparate organisms such as *Drosophila* and mammalians, highlighting the model’s suitability for primary comparative virulence screens (24). Nonetheless, confirmatory evidence in mammalian wound infection models (44, 54) would be warranted. While this was a pathogen-centered study, future investigations would also need to characterize the impact of TSC on fungal interactions with the diverse innate and adaptive immune cell repertoire of mammalian hosts, the local inflammatory environment in infected myocutaneous tissue, and biochemical parameters of wound healing. In addition, comparative studies in immunocompetent and immunosuppressed animals would be needed to account for trauma-induced immune paralysis (1, 55, 56).

Furthermore, our study in a fungal monoinfection model cannot recapitulate the complexity of trauma-related soft tissue infections that are often polymicrobial in nature (1, 9–15). Cross-kingdom interactions of pathogens have been increasingly recognized as key virulence determinants shaping the outcomes of life-threatening infectious diseases (57, 58). Direct physical interaction, interkingdom signaling, altered immunopathology, and competition for nutrients or trace elements are considered to play a driving role in the mutual modulation of bacterial and fungal virulence (57, 59). Interestingly, preliminary results of R. arrhizus and Staphylococcus aureus coinfection studies in our fruit fly model suggest that TSC-induced hypervirulence rather increases in a mixed infection setting (S. Wurster et al., unpublished data), highlighting a need to obtain a more refined understanding of the influence of shear forces on the complex interdependencies in polymicrobial infections.

Despite these limitations, this study, for the first time, blends Mucoralean mechanobiology and pathogenicity and contributes to the understanding of NMM clusters after high-energy trauma events by revealing a hypervirulent phenotype induced by tornadic shear stress. Furthermore, we identified an overarching pathway, whose pharmacological or genetic inhibition fully attenuated increased pathogenicity of shear-challenged Mucorales. This suggests new avenues of adjunct therapeutic interventions in order to improve the detrimental outcomes of NMM in trauma victims.

## MATERIALS AND METHODS

### Fungal culture and shear stress exposure.

The sources and culture conditions of fungal strains used in this study are summarized in Table S1 in the supplemental material. Spores were collected in saline by gently scraping the mycelium with a sterile glass rod. Fungal suspensions were washed twice with sterile saline and spore concentrations were determined using a hemocytometer. Fungal spores were diluted in 30 ml of sterile phosphate-buffered saline (PBS) at a concentration of 1 × 10^7^/ml. The spore suspension was stirred for at least 30 min in a 125-ml Erlenmeyer flask using a Corning PC-353 magnetic stirrer set to maximum speed (∼1,100 rpm). In pilot experiments to establish the optimal shear challenge procedure, spore suspensions were centrifuged at 6,000 × *g* for 30 min or shaken in a 50-ml tube taped to a vortex adaptor at maximum speed for 30 min.

10.1128/mBio.01414-20.4 TABLE S1 Fungal strains used in this study. Download Table S1, DOCX file, 0.03 MB. Copyright © 2020 Wurster et al. 2020 Wurster et al. This content is distributed under the terms of the Creative Commons Attribution 4.0 International license.

### Fungal treatment with calcineurin/hsp90 inhibitors.

For selected experiments, 100 μg/ml cyclosporine (CsA; Sigma-Aldrich) and/or 50 μg/ml tanespimycin (17-*N*-allylamino-17-demethoxygeldanamycin [17-AAG]; Sigma-Aldrich) were added to the spore suspensions either prior to or after TSC. We confirmed in preceding experiments that a concentration of 100 μg/ml CsA does not inhibit growth and morphogenesis of the tested isolates in our IncuCyte NeuroTrack assay. Similarly, the highest noninhibitory concentrations of 17-AAG was determined in preceding experiments using 2-fold serial dilutions.

### *D. melanogaster* infection model.

For Mucorales and *Fusarium* infections, female Oregon^R^ wild-type (WT) D. melanogaster flies were used. Since WT flies are resistant to *Aspergillus* infections (60), A. fumigatus was tested in female *Tl^r632^/Tl^I-RXA^ Drosophila* mutant flies, generated by crossing thermosensitive allele of *Toll* (*Tl^r632^*) flies with null allele of *Toll* (*Tl^I-RXA^*) flies. Standard procedures for the manipulation, housing, and feeding of flies were used as previously described (23, 24). The dorsal side of the thorax of CO_2_-anesthetized flies (7 to 14 days old) was pricked with a size 000 insect pin (Austerlitz) dipped into the spore suspensions. Unless indicated otherwise in the figure legends, 1 × 10^7^/ml spore suspensions were used, and infections were performed within 10 min after termination of the stirring process. Flies were kept at 29°C and transferred into fresh vials every other day. Survival was assessed daily until day 7 postinfection. At least three independent experiments with 20 to 26 flies per experiment and condition were performed on different days.

### IncuCyte assay to monitor fungal morphology, mycelial expansion, and susceptibility to noxious environments.

After termination of the magnetic stirring process to exert tornadic shear stress (TSC), spore suspensions were promptly diluted in RPMI plus 2% glucose at a concentration of 1 × 10^3^ spores per ml. A total of 200 spores were seeded per well of a 96-well flat-bottom plate. For selected experiments, serial dilutions of hydrogen peroxide (H_2_O_2_; Fisher Scientific, final concentration, 0.25 to 64 mM), amphotericin B (0.03 to 16 μg/ml), or posaconazole (0.03 to 16 μg/ml) were added. Phase images were obtained hourly for 24 h at 37°C in the IncuCyte Zoom HD/2CLR time-lapse microscopy system (Sartorius) equipped with an IncuCyte Zoom 10× Plan Fluor objective (Sartorius). The IncuCyte image analysis software was used to quantify mycelial confluence, hyphal length, and branch point numbers as described before (28).

### Hemocyte phagocytosis assay.

*Drosophila* Schneider 2 (S2) cells (Gibco) were cultured in complete Schneider’s medium containing 10% heat-inactivated fetal bovine serum (FBS; Sigma), 0.1% Pluronic F-68 (Gibco), and 50 IU/ml penicillin G plus 50 μg/ml streptomycin sulfate (Gibco). Cells were kept in 75-cm^2^ culture flasks at 28°C and passaged every 3 to 4 days according to the manufacturer’s recommendations. For coculture experiments, S2 cells were centrifuged at 100 × *g* for 5 min, quantified with a hemocytometer, and diluted in fresh complete Schneider’s medium to a concentration of 10^5^/ml. Next, 100-μl aliquots (10^4^ cells) were combined with 10^3^ resting or shear-challenged R. arrhizus FTR1-GFP spores diluted in 100 μl of complete Schneider’s medium in a 96-well flat-bottom plate. The plate was imaged hourly (phase and green fluorescence, 400-ms acquisition time) in the IncuCyte ZOOM time-lapse microscopy system for 24 h at 28°C. Hyphal length and branch point numbers per mm^2^ were quantified by NeuroTrack analysis as described before (28).

### Measurement of *Rhizopus*-induced host cell damage.

*Rhizopus*-induced A549 cell damage was quantified using a Pierce LDH assay with slight modifications to the manufacturer’s protocol. Briefly, A549 cells were grown in 96-well tissue culture plates for 18 to 24 h in F12k medium with l-glutamine plus 10% FBS. Spores from *R. delemar* strain 99-880 (a clinical isolate obtained from a patient with rhino-orbital mucormycosis) were washed, resuspended in PBS, and then subjected to TSC by magnetic stirring or allowed to sit without stirring (control). Thereafter, spores were added to A549 cells at three different concentrations (2 × 10^6^, 4 × 10^5^, or 8 × 10^4^ spores per well). After 24 and 48 h of incubation at 37°C, 50 μl of the cell culture supernatant was collected from each well and transferred to a 96-well plate to assay for LDH activity. LDH is a cytosolic enzyme that is released into the cell culture medium upon cell membrane damage. The amount of extracellular LDH is proportional to the amount of cell damage. Lysis buffer was added to all infected wells and incubated for 45 min at 37°C. After lysis, 50 μl of cell culture supernatant was transferred to another 96-well plate and used for the LDH assay kit per protocol. LDH release was calculated as previously described (61).

### Fungal RNA isolation.

A total of 2 × 10^7^ spores were mixed with 1 ml of RNA*later* RNA stabilization reagent (Qiagen) and centrifuged at 17,000 × *g* for 5 min. The supernatant was discarded, and spores were resuspended in 500 μl of RLT buffer (Qiagen) supplemented with 1% β-mercaptoethanol. Bead beating was performed for 2 × 30 s by using a Mini Beadbeater (Biospec Products) and UltraClean microbial RNA bead tubes (MoBio), followed by a 3-min incubation step at 56°C. Thereafter, RNA was isolated using the RNeasy Plant minikit (Qiagen) according to the manufacturer’s instructions. RNA yield and purity were determined with a NanoDrop spectrophotometer (Thermo Scientific) and an Agilent 2100 bioanalyzer.

### RNA sequencing and gene expression analysis.

RNA-seq libraries (strand-specific, paired end) were generated from total fungal RNA by using a TruSeq RNA sample prep kit (Illumina). Seventy-five nucleotides of the sequence were determined from both ends of each cDNA fragment using the HiSeq 4000 platform (Illumina). Sequencing reads were aligned to the reference *R. delemar* 99-880 genome using HISAT (62), and alignment files were used to generate read counts for each gene. Statistical analysis of differential gene expression was performed using the DEseq package from Bioconductor (63). A gene was considered differentially expressed if the FDR value for differential expression was <0.05. The RNA-seq analysis was performed in biological triplicate.

### Statistics.

Data analysis was performed using Microsoft Excel 2013 and GraphPad Prism 7.03. A log rank test (Mantel-Cox test) was used to compare survival curves. For *in vitro* readouts, a two-sided *t* test or a one-way analysis of variance with Tukey’s *post hoc* test was used for significance testing depending on the data format. A *P* value of <0.05 was considered significant.

### Data availability.

The raw sequencing reads from this study have been submitted to the NCBI sequence read archive (SRA) under BioProject accession no. PRJNA632748. The data are also available upon request.
