# Supplementary figures and images for: Tornadic Shear Stress Induces a Transient, Calcineurin-Dependent Hypervirulent Phenotype in Mucorales Molds
Source: mBio. 2020 Jun 30;11(3):e01414-20. doi: 10.1128/mBio.01414-20 (PMC7327176; doi:10.1128/mBio.01414-20)

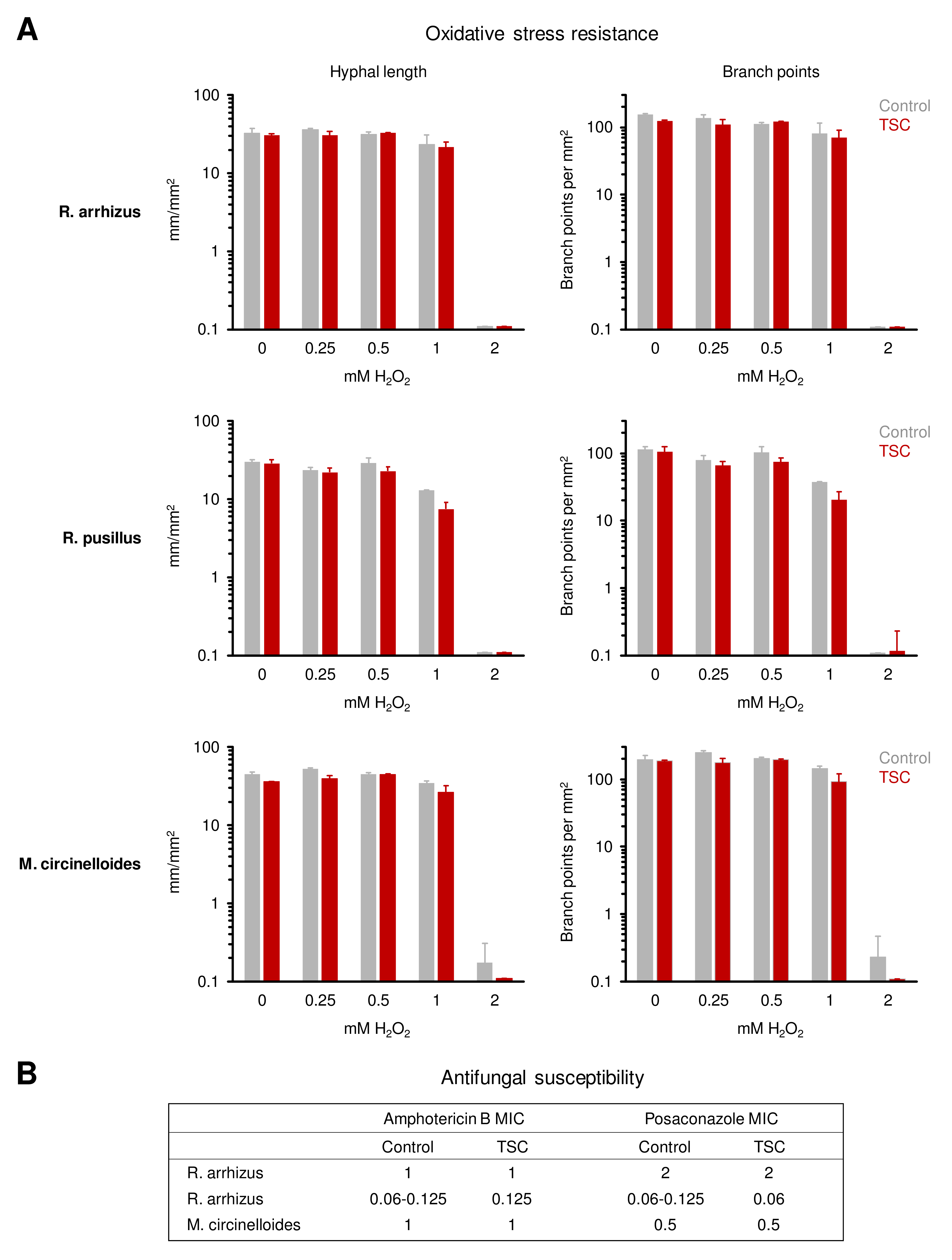

Supplement: FIG S1 [file mBio.01414-20-sf001.tif]

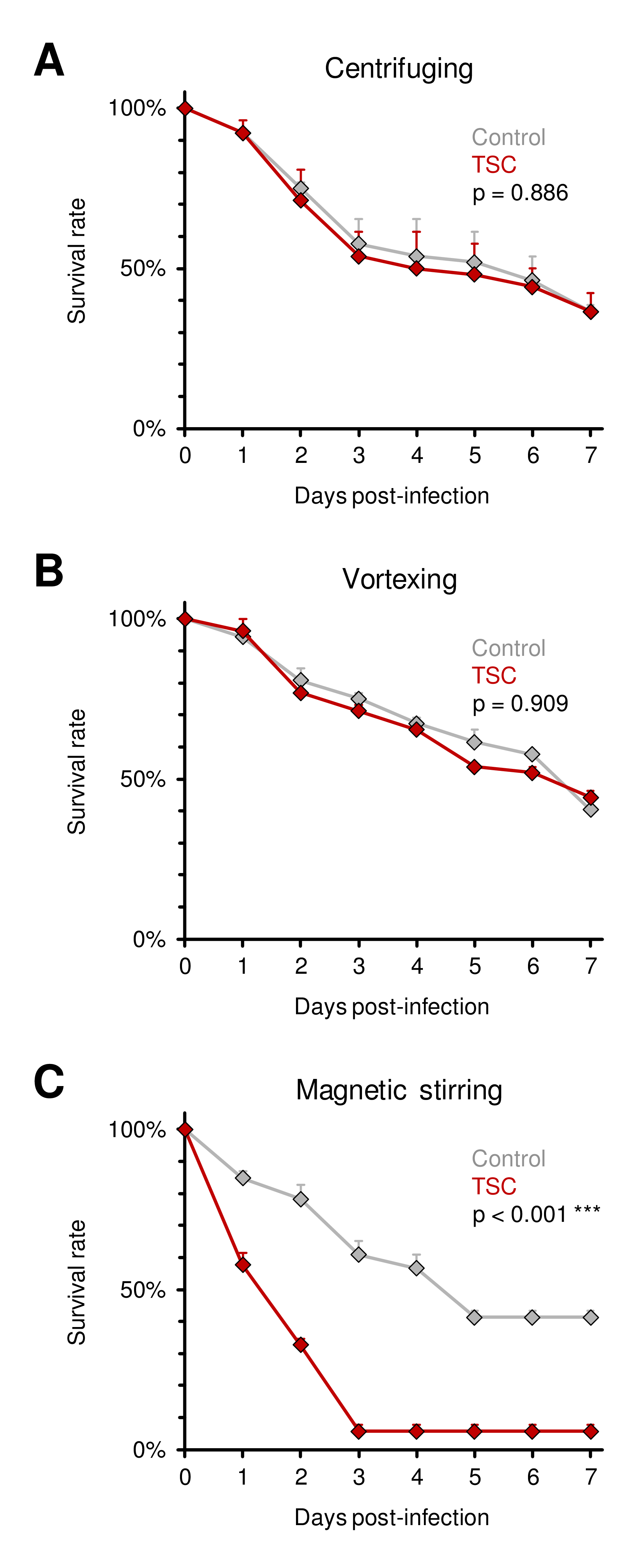

Supplement: FIG S2 [file mBio.01414-20-sf002.tif]

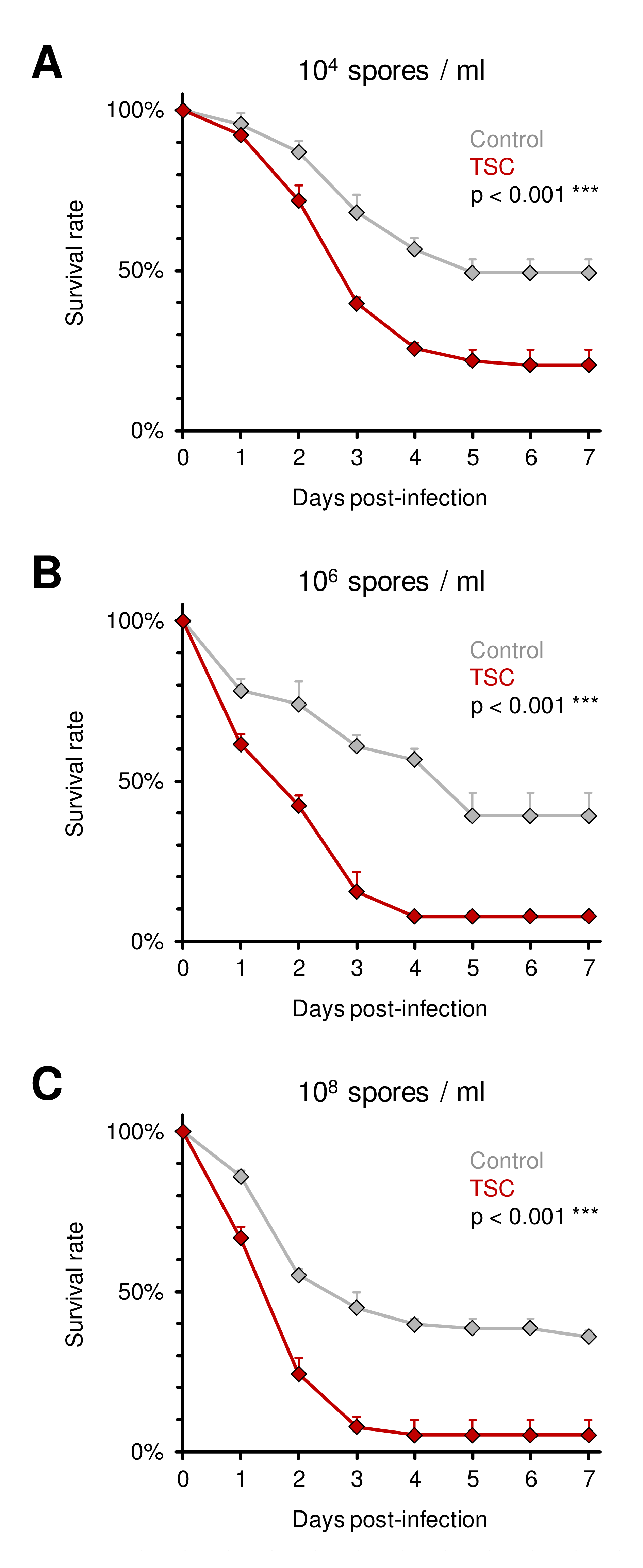

Supplement: FIG S3 [file mBio.01414-20-sf003.tif]
