# Supplementary material for: Tornadic Shear Stress Induces a Transient, Calcineurin-Dependent Hypervirulent Phenotype in Mucorales Molds
Source: mBio. 2020 Jun 30;11(3):e01414-20. doi: 10.1128/mBio.01414-20 (PMC7327176; doi:10.1128/mBio.01414-20)
Supplement: TABLE S1 [file mBio.01414-20-st001.docx]

Table S1. Fungal strains used in this study.

| **Species** | **Strain** | **Source/reference** | **Agar/medium** | **Temp.** | **Culture**  **duration** |
| --- | --- | --- | --- | --- | --- |
| *Rhizopus arrhizus* | Ra-749 | Clinical isolate (S1) | Yeast extract agar | 37 ˚C | 2-3 days |
| *Rhizopus arrhizus* | Ra-969 | Clinical isolate (S1) | Yeast extract agar | 37 ˚C | 2-3 days |
| *Rhizopus arrhizus* | FTR1-GFP-  *R. arrhizus* | (S2) | Yeast nitrogen base agar + complete supplement mixture minus uracil | 37 ˚C | 3-5 days |
| *Rhizopus (arrhizus*  *var.) delemar* | RA 99-880 /  ATCC MYA-4621 | American Type  Culture Collection | Peptone dextrose agar | 37 ˚C | 3-5 days |
| *Rhizomucor pusillus* | Rp-449 | Clinical isolate (S1) | Yeast extract agar | 37 ˚C | 2-3 days |
| *Mucor circinelloides* | Mc-518 | Clinical isolate (S1) | Yeast extract agar | 30 ˚C | 2-3 days |
| *Mucor circinelloides* | R7B | (S3-S4) | Yeast peptone glucose agar | 30 ˚C | 5 days |
| *Mucor circinelloides* | *cnaAΔ* (MSL9) | (S3-S4) | Yeast peptone glucose agar | 30 ˚C | 5 days |
| *Mucor circinelloides* | *cnaBΔ* (MSL22) | (S4) | Yeast peptone glucose agar | 30 ˚C | 5 days |
| *Mucor circinelloides* | *cnbRΔ* (MSL8) | (S3-S4) | Yeast dextrose agar | 30 ˚C | 2 days |
| *Apophysomyces*  *trapeziformis* | CBS 125534 | CBS-KNAW Fungal Biodiversity Centre | Minimal medium | 37 ˚C | 5 days |
| *Aspergillus fumigatus* | Af-293  ATCC MYA-4609 | American Type  Culture Collection | Yeast extract agar | 37 ˚C | 2-3 days |
| *Fusarium solani* | Fs-001 | Clinical isolate (S1) | Yeast peptone glucose liquid medium | 30 ˚C | 3-5 days |

(S1) Isolates were obtained from cancer patients at the University of Texas MD Anderson Cancer Center, Houston, Texas, USA.

(S2) Ibrahim AS, Gebremariam T, Lin L, Luo G, Husseiny MI, Skory CD, Fu Y, French SW, Edwards JE Jr, Spellberg B. The high affinity iron permease is a key virulence factor required for Rhizopus oryzae pathogenesis. Mol Microbiol. 2010; 77(3):587-604.

(S3) Lee SC, Li A, Calo S, Heitman J. Calcineurin plays key roles in the dimorphic transition and virulence of the human pathogenic zygomycete Mucor circinelloides. PLoS Pathog. 2013; 9(9):e1003625.

(S4) Lee SC, Li A, Calo S, Inoue M, Tonthat NK, Bain JM, Louw J, Shinohara ML, Erwig LP, Schumacher MA, Ko DC, Heitman J. Calcineurin orchestrates dimorphic transitions, antifungal drug responses and host-pathogen interactions of the pathogenic mucoralean fungus Mucor circinelloides. Mol Microbiol. 2015; 97(5):844-65.
